# Metabolic Syndrome, Inflammation, and Cancer

**DOI:** 10.1155/2017/8259356

**Published:** 2017-07-06

**Authors:** Yong Wu, Yunzhou Dong, Shengzhong Duan, Donghui Zhu, Lin Deng

**Affiliations:** ^1^Division of Cancer Research and Training, Department of Internal Medicine, Charles R. Drew University of Medicine and Science, Los Angeles, CA, USA; ^2^David Geffen UCLA School of Medicine and UCLA Jonsson Comprehensive Cancer Center, University of California, Los Angeles, CA, USA; ^3^Department of Surgery, Boston Children's Hospital and Harvard Medical School, Boston, MA 02115, USA; ^4^Laboratory of Oral Microbiology, Shanghai Research Institute of Stomatology, Shanghai Key Laboratory of Stomatology, Ninth People's Hospital, Shanghai Jiao Tong University School of Medicine, Shanghai 200011, China; ^5^University of North Texas, Denton, TX, USA; ^6^Dana-Farber Cancer Institute, Harvard Medical School, Boston, MA 02115, USA

The inflammatory condition associated with overweight/obesity represents a triggering factor in the pathogenesis of the metabolic syndrome and primarily contributes to the related pathological outcomes. Stimuli including overnutrition, physical inactivity, and ageing might lead to oversecretion of proinflammatory cytokine, ultimately resulting in insulin resistance, diabetes, and its cardiovascular complications. Furthermore, inflammation has also been associated with several types of cancers by influencing growth, apoptosis, and proliferation of cancer and stromal cells.

Previous studies propose that the metabolic syndrome may play an imperative role in the initiation, progression, and poor prognosis of some tumors. Currently, the causal association between the metabolic syndrome and cancer is more commonly recognized; nevertheless, the precise mechanisms mediating this relationship remain poorly understood. It has become evident that the inflammatory condition associated with metabolic syndrome contributes to the development and progression of cancer. Ascertaining the link between the metabolic syndrome and cancer, the role of inflammation in these diseases and identification of new therapeutic targets are of great significance. This special issue contributes original research papers and review articles that motivate the continuous efforts to comprehend the mechanisms, production, and management related to cancers associated with the metabolic syndrome.

In this special issue, the link between the metabolic syndrome and cancer was extensively discussed. Both cancer and diabetes are associated with abnormal lactate metabolism, and high lactate level is the crucial biological feature of these diseases. On the other hand, high lactate results in a higher insulin-resistant status and a more malignant phenotype of cancer cells, favoring diabetes and cancer development. Considering an interactive relationship between diabetes and cancers, the role of high lactate production in diabetes and cancer interaction should not be neglected. Understanding the molecular mechanisms underlying metabolic remodeling of diabetes- and cancer-related signaling would provide novel preventive and therapeutic approaches for diabetes and cancer treatment. In addition, S. Harlid et al. interrogated the association between the metabolic syndrome and colorectal cancer risk in human subjects. Out of 178 inflammatory and cancer biomarkers, 12 proteins were associated with the metabolic syndrome and/or its components. FGF21, one of the 12 proteins, was also associated with an increased risk for colorectal cancer, exemplifying the intimate relationship between the metabolic syndrome and cancer.

In the present special issue, the anti-inflammatory role and tissue-/organ-protective effects of different reagents and drugs were also studied. Y. Gao et al. revealed that H2 saline has a protective effect against doxorubicin-induced cardiotoxicity and hepatotoxicityin rats by inhibiting inflammation and apoptosis. K. Su et al. demonstrated that FTY720, a new chemical substance derived from the ascomycete *Isaria sinclairii*, is able to attenuate sphingosine-1-phosphate- (S1P-) induced podocyte damage via reducing inflammatory cytokines. Using system pharmacological analysis, X. Shen et al. suggested that combined application of Bupleuri Radix and Scutellaria Radix not only directly inhibit the synthesis and release of inflammatory cytokines but also have potential therapeutic effects against inflammation-induced pain. Additionally, a combination therapy of these two drugs exhibited systemic treatment efficacy and provided a theoretical basis for the development of drugs against inflammatory diseases. Y. Dong et al. elicited the role of lysophosphatidic acid (LPA), a naturally occurring bioactive phospholipid, in the regulation of cell survival and apoptosis in HeLa cells. Under pathological conditions, high concentration of LPA triggers apoptosis by the upregulation of TNFR21 (DR6) expression, one of the death receptors in inflammation, which solved a controversial question in different literatures. In clinical studies, X. Liu et al. revealed that the antidiabetic drugs metformin, sitagliptin, and pioglitazone have an anti-inflammatory role in atherosclerosis, suggesting a therapeutic role of these drugs in preventing diabetic atherosclerosis, and furthermore, combinational therapy was beneficial to reduce atherosclerosis. Another clinical study by X. Ding et al. suggest that the components of the metabolic syndrome may associate with the higher risks of thyroid nodule (TN) in women than in men. Furthermore, S. S. Chung et al. proposed that proinflammatory cytokines induced cancer cell invasiveness and this was mediated by a signal transducer and activator of transcription 3- (STAT3-) regulated mechanism in colorectal cancer cells. Their data suggest that withaferin A could be a promising anticancer agent that effectively inhibits the progression of colorectal cancer.

We anticipate that the present special issue will not only be valuable to the extensive readership by providing insights into novel and chief aspects associated with inflammation and its mediators in the context of the metabolic syndrome and cancer but also inspire innovative research ideas and revolutionized therapeutic strategies in this field.

