# Corrigendum to “Metabolic Syndrome, Inflammation, and Cancer”

**DOI:** 10.1155/2017/6598540

**Published:** 2017-12-21

**Authors:** Yong Wu, Yunzhou Dong, Shengzhong Duan, Donghui Zhu, Lin Deng

**Affiliations:** ^1^Division of Cancer Research and Training, Department of Internal Medicine, Charles R. Drew University of Medicine and Science, Los Angeles, CA, USA; ^2^David Geffen UCLA School of Medicine and UCLA Jonsson Comprehensive Cancer Center, University of California, Los Angeles, CA, USA; ^3^Department of Surgery, Boston Children's Hospital and Harvard Medical School, Boston, MA 02115, USA; ^4^Laboratory of Oral Microbiology, Shanghai Research Institute of Stomatology, Shanghai Key Laboratory of Stomatology, Ninth People's Hospital, Shanghai Jiao Tong University School of Medicine, Shanghai 200011, China; ^5^University of North Texas, Denton, TX, USA; ^6^Dana-Farber Cancer Institute, Harvard Medical School, Boston, MA 02115, USA

In the article titled “Metabolic Syndrome, Inflammation, and Cancer” [1], an Acknowledgments section should be added as follows.
